# Anatomical Adaptations of Halophyte Leaves (*Nitraria retusa* [Forsskal] Asch. and *Atriplex halimus* L.) in Response to Cement Dust Pollution in Arid Environments

**DOI:** 10.3390/life15010061

**Published:** 2025-01-07

**Authors:** Nouha Krir, Mounira Mkaddem Guedri, Mehrez Romdhane, Manel Abdullah Alshaqha

**Affiliations:** 1 Laboratory of Energy, Water, Environment and Process (LR18ES35), National Engineering School of Gabes, University of Gabes, Rue Omar Elkhattab-ZRIG-6029, Gabes 6072, Tunisia; nouha.krir@gmail.com (N.K.); mehrez.romdhane@univgb.tn (M.R.); 2 Biology Department, College of Science, King Khalid University [KKU], Abha 61413, Saudi Arabia; malshgha@kku.edu.sa

**Keywords:** bioindicator, cement dust, environmental pollution, phytoremediation, *Nitraria retusa*, *Atriplex halimus*, leaf anatomy, heavy metals

## Abstract

This study investigates the anatomical adaptations of leaves from two halophyte species, *Nitraria retusa* (Forsskal) Asch. and *Atriplex halimus* L., in response to pollutants from a cement factory and human activities. In industrial areas, these plants absorb pollutants through their leaf surfaces, including Cu, Zn, and Pb. The two species were examined for anatomical changes under air pollution, and key factors including leaf blade thickness, palisade parenchyma cell height, spongy parenchyma cell diameter, epidermal characteristics, and stomatal traits were assessed. Under pollution, the leaves displayed smaller and denser stomata and idioblasts in the palisade and spongy parenchyma. These anatomical responses suggest that *N. retusa* and *A. halimus* could be effective bioindicators for detecting cement dust pollutants. Their leaf relative water content (RWC) exhibited a range of values: 70.1% and 87% for *N. retusa* and 64.8% to 74.2% for *A. halimus* on the highly polluted site (S1) and the control site (S4), respectively. Notably, a statistically significant site effect was observed (*p* > 0.01), confirming previous studies, and indicating reduced leaf relative water content (RWC) values in plants exposed to heavy metals like Cd and Pb. Heavy metals can lead to mineralization by binding to cell walls, altering their physicochemical properties and plasticity. Furthermore, significant correlations between specific heavy metals and histological parameters in *A. halimus* leaves indicated potential interactions between metal composition and leaf structure, highlighting their role in modulating anatomical adaptations. The correlation of leaf thickness, upper epidermal thickness, and stomatal density with Zn and Pb levels underlines the importance of these anatomical features in heavy metal accumulation and retention in plant tissues.

## 1. Introduction

Air pollution, mainly in the form of particulate matter, is a significant global issue affecting developed and developing countries, with harmful effects on human health and the environment. Among the pollutants of greatest concern are heavy metals (HMs), such as lead (Pb), cadmium (Cd), and zinc (Zn), which are released from anthropogenic sources like industrial activities, energy production, and vehicular traffic [1,2,3,4]. Vehicle emissions contribute to the contamination of air, soil, and water through combustion, wear of vehicle components, and the resuspension of road dust [5,6,7]. Additionally, cement production and construction activities release particulate matter and other pollutants into the atmosphere, thus damaging the environment and human health. When cement dust settles on plant leaves, it obstructs light absorption, reduces photosynthesis, and leads to anatomical changes in the leaves, including reduced stomatal density and alterations in stomatal size and shape, disrupting the plant’s physiological processes [8,9]. This interference not only hampers plant growth but also reduces their efficiency in mitigating atmospheric CO_2_ levels, thereby exacerbating global warming. Furthermore, the deposition of cement dust can lead to the accumulation of toxic substances, including heavy metals, in soils and water sources, posing additional risks to ecosystems. Addressing these challenges requires a comprehensive understanding of the interactions between industrial pollution and environmental health [10]. Over the past few decades, plants have gained attention as potential bioindicators for monitoring air pollution due to their ability to accumulate pollutants through atmospheric deposition and root uptake, depending on external environmental conditions and their specific physiology [11,12,13]. Despite the recognition of this potential, the use of plants for pollution monitoring in regions like Gabes, an area of Tunisia with high levels of pollution due to industrial and vehicular emissions, has not been extensively explored. The Gabes region, located in southeastern Tunisia, is grappling with serious environmental challenges stemming from its fragile ecosystem, inadequate urban planning, and rapid industrial growth. A major contributor to these issues is the Gabes cement factory which releases significant quantities of heavy metals and toxic gases, including NOx and SO_2_, as byproducts of phosphate processing [14]. For four decades, approximately 12,000 tons of untreated phosphogypsum, a byproduct of phosphate fertilizer production, have been dumped into the environment daily. This waste is rich in fluoride and contains high levels of heavy metals such as copper (Cu), zinc (Zn), cadmium (Cd), lead (Pb), and chromium (Cr) [14]. Furthermore, the region is heavily affected by air pollution from vehicular traffic, as many cars still rely on leaded fuel, further degrading the local environment [1].

This study investigates the potential of two native halophyte species, *Atriplex halimus* L. and *Nitraria retusa* (Forsskal) Asch., to serve as bioindicators of cement dust pollution under the challenging climatic conditions of Gabes. *Atriplex halimus* is widely regarded for its ability to thrive in saline soils, making it a crucial species for soil reclamation and desertification control in arid regions [15,16]. Similarly, *Nitraria retusa* plays an essential role in stabilizing coastal and inland ecosystems, especially in areas prone to erosion and salinization [15,17,18]. Both species, known for their salt tolerance and ecological significance, contribute to the resilience of the local environment, supporting biodiversity and improving soil structure. Additionally, the leaves of these species are rich in bioactive compounds, making them valuable in traditional medicine, particularly for the treatment of inflammatory diseases and digestive disorders [19,20]. Their ability to accumulate pollutants such as heavy metals and particulate matter further enhances their potential as indicators of environmental pollution. Given the increasing industrial activities in the Gabes region, these halophytes could offer a cost-effective and environmentally friendly method for monitoring cement dust pollution, thus contributing to both ecological conservation and public health protection.

By assessing the ecophysiological and anatomical responses of their leaves to cement dust, this study evaluates their effectiveness in monitoring atmospheric pollution and contributes to developing sustainable pollution-tracking methods in urban environments.

## 2. Material and Methods

### 2.1. Study Sites and Sampling

**Figure 1:**
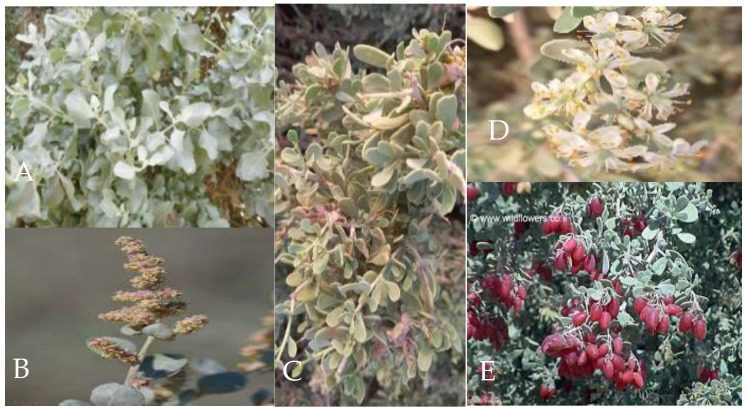
A. *halimus* (**A**,**B**) and *N. retusa* (**C**–**E**) growing wild in Gabes city, southeastern Tunisia).

Gabes city (33°53′ N, 10°07′ E; southeastern Tunisia) is under pressure from urbanization and industrialization, two major pollution sources. In addition to its vulnerable arid ecosystem, the region suffers from bad urban management and rapid road traffic. On the other hand, it contains an important industrial complex, which is a significant source of environmental pollution. *A. halimus* and *N. retusa* leaves were collected in the southeast of Gabes city (in the spring of 2021) (Figure 1) in the Elhemma (S1), Tebelbou (S2), Mareth (S3), and Matmata (S4) sites located 2, 30, 45, and 6 km from the industrial complex, respectively (Table 1). All four sites exhibit the same meteorological, air, and soil characteristics [21].

### 2.2. Heavy Metal Content

HM was detected according to the protocol described by [22].Samples were collected from each site and individually preserved. Plants were divided into belowground roots and aboveground shoots and thoroughly washed with distilled water to eliminate dust and other solid particles. Samples were then dried at 80 °C for 24 h, ground, and homogenized through a 0.2 mm sieve. Dry samples of 0.5 g were ashed at 550 °C in a muffle furnace for 3 h and digested using 10 mL of 2.8% HNO_3_.

The solutions were analyzed for Zn, Pb, copper (Cu), and Cd using a flame atomic absorption spectrophotometer (GBC scientific Avanta spectrophotometer model AA, Draper QLD 4520, Australia). HM concentrations in the dry plant material were expressed in mg kg^−1^, and all measurements were carried out in triplicate.

### 2.3. Relative Water Content

The method outlined by Scippa et al. (2004) was followed. The relative water content (RWC) was calculated using the following formula:

RWC (%) = (FW − DW) × 100/(TW − DW)

FW—fresh leaf weight; DW—dry leaf weight; and TW—turgid leaf water.

### 2.4. Anatomical Studies

#### 2.4.1. Histology

The histological study of *N. retusa* and *A. halimus* leaves was conducted using Johansen’s method (1940) [23]. Samples were fixed in an FAA solution, progressively dehydrated with ethanol and TBA mixtures, and embedded in paraffin. Transverse sections obtained with a microtome were mounted on glass slides, deparaffinized, and stained with safranin and fast green to highlight anatomical structures. After final dehydration and mounting with Canada balsam, the sections were observed under an optical microscope, allowing detailed analysis and imaging at ×100 and ×400 magnifications.

#### 2.4.2. Anatomical Analysis

We employed Nikon’s Z50 II biometric software to measure the dimensions of epidermal cells, stomata, and parenchyma cells. This tool allowed for the precise measurement and analysis of anatomical features in the prepared sections, ensuring accurate quantification and enhanced clarity of structural observations, contributing to the robustness of the histological analysis. One section from each leaf was examined, and the following anatomical parameters were quantified: leaf thickness (µm), height of palisade cells (µm), diameter of spongy cells (µm), height and width of upper epidermal cells (µm), and stomatal index following the method described by [24].

### 2.5. Statistical Analysis

All data were analyzed using SPSS statistical software Version 17.0 (2008). The results are presented as the mean ± standard error of the mean (SEM) of triplicate measurements. A one-way analysis of variance (ANOVA) was used to compare differences among study sites. Duncan’s post hoc test was performed to compare the means at *p* < 0.05 for statistical significance. The correlation between the leaf HM content of the two species and the values of anatomical characters was assessed by Pearson’s correlation coefficient (*p* < 0.05) (SSP, 2023) [25].

## 3. Results and Discussion

### 3.1. HMC

HM concentrations (Zn, Cu, Pb, and Cd) (mg kg^−1^ DW) in leaf samples varied within a species (Table 2). High levels of all four elements were found in samples collected on S1, while the lowest concentrations were recorded on S4 for the two species. Zn, Pb, and Cu are minor trace elements required in small amounts for plant nutrition, development, and several metabolic reactions [26]. However, at high concentrations, they can become lethal [27]. Our results indicated that, on all study sites, micronutrient levels did not exceed toxic levels (Table 3).

HM concentrations (Zn, Cu, Pb, and Cd) (mg kg^−1^ DW) in leaf samples varied within a species (Table 2). High levels of all four elements were found in samples collected on S1, while the lowest concentrations were recorded on S4 for the two species. Zn, Pb, and Cu are minor trace elements required in small amounts for plant nutrition, development, and several metabolic reactions However, at high concentrations, they can become lethal Our results indicated that, on all study sites, micronutrient levels did not exceed toxic levels (Table 3).

*N. retusa* leaves had significantly higher Pb and Cd mean concentrations than *A. halimus* (Table 3), making it suitable for phytoextraction from the soil. Lead pollution is primarily caused on a large scale by emissions from motor vehicles that use leaded gasoline [28]. Typical Pb concentrations in plants range from 5 to 10 mg kg^−1^, with toxic concentrations from 30 to 300 mg kg^−1^ [29,30]. *N. retusa* exhibited toxic Pb levels and the highest mean concentration of Cd in leaves (22.66 mg kg^−1^). In contrast, *A. halimus* had the lowest. The two species had Cd concentrations far below the phytotoxic level of 5 mg kg^−1^ [31]. However, *A. halimus* has the highest mean concentrations of Zn and Cu in leaves. Typically, Zn and Cu concentrations are <150 and 30 mg kg^−1^, respectively, in these species [30,32]. In the present study, Zn concentrations were within the normal range, while a Cu concentration higher than the critical value was observed in *A. halimus* leaves.

The rise in the concentration of specific metals could be related to anthropogenic sources. In our case, such a source refers to the cement factory and the chemical group pollution in Gabes City. These metals are dispersed next to the studied plant species and can induce various alterations in the soil’s physicochemical properties.

### 3.2. Leaf RWC

**Figure 2:**
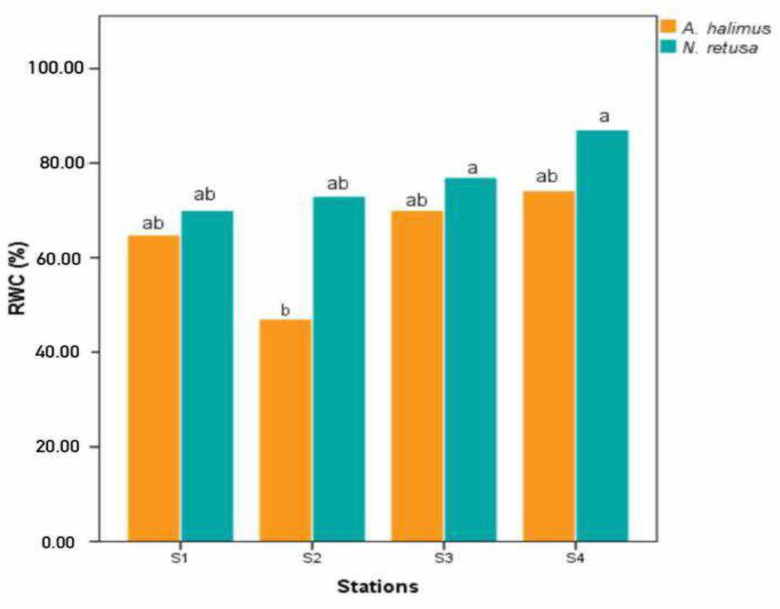
The leaf relative water content (RWC) comparison of *A. halimus* and *N. retusa* at the five sites studied. Different letters on the bars indicate significant differences at *p* < 0.05.

The leaf RWC exhibited a range of values, 70.1% to 87% for *N. retusa* and 64.8% to 74.2% for *A. halimus* on the highly polluted site (S1) and the control site (S4), respectively. As illustrated in Figure 2, notably, there was a statistically significant site effect (*p* > 0.01) for either species, according to ANOVA statistical analysis (Figure 2). This reduction in RWC% from the control site (S4) to the most polluted site (S1) aligns with previous findings, which reported lower RWC values in plants treated with Cd [20]. Some HMs, such as Pb, can cause mineralization by binding to the cell wall, changing its physicochemical properties and plasticity [9,33]. In 2016, Rucinska-Sobkowiak [34] observed that soil HM concentrations typically do not reach levels that result in osmotic disruptions in plants. The entry of water into plant roots is indirectly regulated by various factors, which, in turn, are influenced by metal presence. De Silva et al. [35] suggested a disrupted plant water status induced by HMs may be attributed to the inhibition of hydraulic conductivity within plants, stemming from a gradual reduction in the proportion of xylem available for water transport. HM stress in pea plants adversely impacted their hydraulic architecture by promoting water loss through transpiration, which was associated with reduced leaf size, lamina thickness, and mesophyll intercellular spaces [36]. Other studies have also indicated that HMs can influence stomatal density and the ostiole, which are central in regulating water status [37].

### 3.3. Leaf Anatomical Features

**Figure 3:**
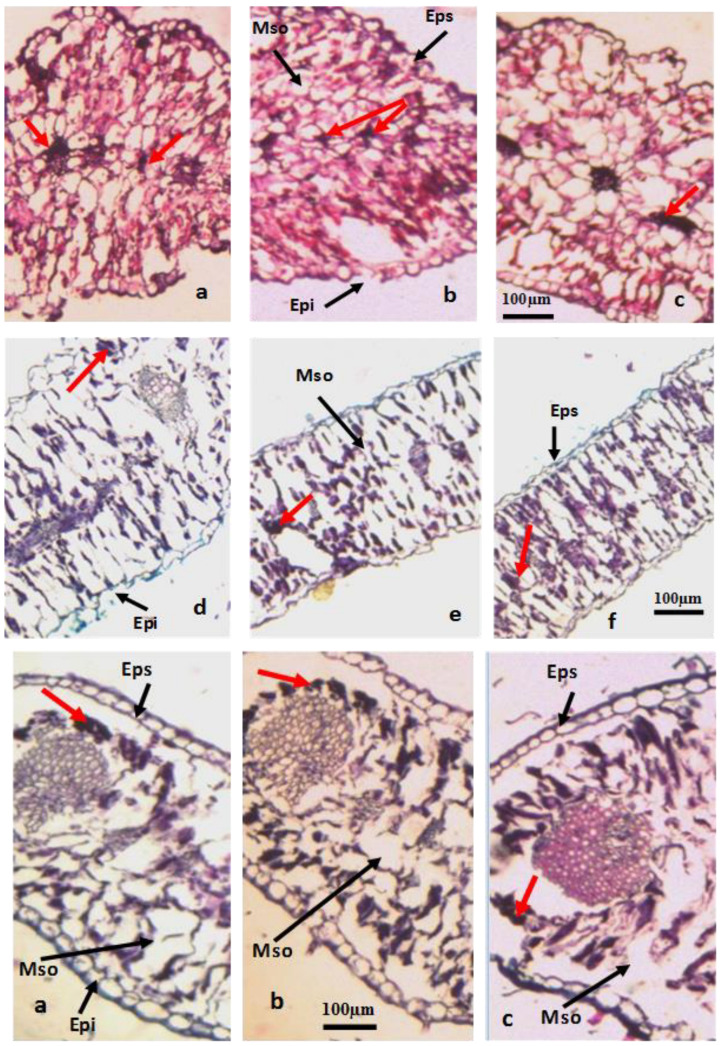
Transverse sections of leaves from *Atriplex halimus* (**above** **a**–**f**) and *Nitraria retusa* (**below** **a**–**c**) and collected from study sites. Eps: upper epidermis; Epi: lower epidermis; Mso: mesophyll. Red arrows indicate idioblasts storing pollutant deposits.

Foliar stomata represent the primary pathway for aerosol entry. Environmental stresses induce structural and ultrastructural alterations in leaves, enabling them to withstand the effects of atmospheric pollutants [8].In fact, the leaves of the examined plant species exhibited notable modifications in their anatomical characteristics (Figure 3).

#### 3.3.1. Foliar Epidermal Thickness

The total leaf thickness of *N. retusa* decreased at the cement factory site (S1) (281.25 µm) compared to the control (S4) (397.5 µm) (Table 2). A significant increase was observed in the leaf thickness of *A. halimus*, from 471.66 µm on S1 to 355.83 µm on S4 (Table 2). The upper epidermis of the leaf appeared to exhibit resistance to the effects of pollution in the two species. Its thickness decreased by 40.35% in *A. halimus* and 19.44% in *N. retusa* on S1 compared to S4. However, the thickness of the lower epidermis was less affected by pollution in the two species; in *A. halimus*, it was 8.79 µm on S1 and 9.06 µm on S4. Similarly, in *N. retusa*, the lower epidermal thickness was 12.70 µm on S1 and 16.7 µm on S4 (Matmata). The upper and lower epidermal cells thickened in response to pollution in *A. halimus*. The presence of thickened epidermal layers aligns with observations made in *Trifolium montanum* L. [38], *Brachiaria decumbens* Stapf. [39], and *Betula pendula* Roth. [40] exposed to various pollution factors. The increase in epidermal thickness reduces the ingress of toxic gases into the leaf [41]. The slight non-significant reduction in the lower epidermis thickness we observed may be explained by the fact that the epidermal features of these leaves remained unaffected by environmental pollution. The atmospheric pollutant levels may not have surpassed the threshold required to alter the leaf epidermis [42]. The reduced thickness of the upper epidermis could be elucidated by [43,44], who observed increased transpiration in plants subjected to heavy dust deposition from inert particles [45].

#### 3.3.2. Mesophyll Cells Size

The size of mesophyll cells significantly decreased in response to pollution by 32.51% in *N. retusa* and 40.79% in *A. halimus* on S1. The total leaf and mesophyll thickness exhibited varied responses depending on the studied species and the pollution level. A notable increase was observed in the leaf and mesophyll thickness of *A. halimus*. This finding aligns with the studies conducted by [46], which demonstrated an increased leaf thickness in *Phaseolus vulgaris* L. exposed to high CO_2_ concentrations. Concurrently, [40] observed an increased leaf thickness in *Betula pendula* Roth. under industrial pollution in urban areas [47].

Foliar succulence in relation to leaf thickness can be regarded as an ecologically significant factor in mitigating abiotic stresses [48]. Gaseous pollutants primarily gain entry through the stomatal pores concentrated on the lower surface of the leaf. Upon traversing the stomata of the lower epidermis, the toxic gas promptly accumulates in the spongy mesophyll. Due to its larger cells and intercellular spaces, this tissue presents less resistance to gaseous exchange than the palisade parenchyma. Consequently, it is highly susceptible to damage inflicted by air pollutants [49]. Furthermore, Ref. [50] postulated that a high palisade/spongy parenchyma ratio is an indicator of a species’ resistance to air pollution.

Our measurements revealed a reduction in the total leaf thickness and mesophyll tissue in *N. retusa*. This finding is consistent with the results reported by [38], which indicated that *Tanacetum vulgare* L. displayed a reduced total leaf thickness under HM pollution. Furthermore, a study conducted by [51] on a legume species, *Cajanus cajan* (L.) Huth, demonstrated a decrease in the size of palisade and spongy parenchyma cells. Similarly, Zn hyperaccumulation leads to a reduction in the size of mesophyll cells in *Arabidopsis halleri* (L.) [4]. On the other hand, the leaf curling in *A. halimus* may be a strategy to reduce the transpiration surface area. Stomata are maintained within a humid microclimate, mitigating the risk of desiccation [48]. The enlargement of bulbous cells in *A. halimus* subjected to cement factory pollution enhances this curling phenomenon. Ref. [39] similarly reported an increase in the size of bulbous cells in *Brachiaria decumbens* Stapf. exposed to Cd. Likewise, this thickening of bulbous cells has been observed in *Cenchrus ciliaris* L. cultivated in soil contaminated by HMs [52].

#### 3.3.3. Cribro-Vascular Bundle Diameter

In *N. retusa*, the diameter of cribro-vascular bundles exhibited no statistically significant variation between S1 (92.5 µm) and S4 (90 µm). In *A. halimus*, a non-significant increase in the cribro-vascular bundle diameter was observed on the polluted sites, with 100 µm on S1 against 94.16 µm on S4. The vascular tissue size exhibited a non-significant increase in the two plants exposed to pollution effects. This observation is consistent with the findings of [53], who documented an expansion in the surface area of the vascular bundle zone in *Hibiscus rosa-sinensis* L. under industrial pollution influence. Ref. [54] established a direct correlation between the vascular bundle area and the transportation of water and soil nutrients. These transport mechanisms are essential under reduced moisture availability.The presence of an expanded phloem further facilitates the transportation of photosynthetic products within plants [55]. The increased vascular tissue density can be considered a comprehensive indicator of plant survival. It is potentially correlated with the sustained growth observed in the presence of pollution and abiotic stress factors, as suggested by [56,57].

#### 3.3.4. Stomatal Density and Size

Stomatal density and size are critical parameters for assessing plant responses to environmental stress. The results revealed a significant increase in stomatal density for the two species, with 52.75 for *N. retusa* and 75.475 for *A. halimus* on S1. Regarding stomatal size, a significant decrease (*p* < 0.05) was observed for the two species on polluted sites compared to S4. *A. halimus* exhibited the largest stomatal size (41.8 µm on S1), followed by *N. retusa* (33.67 µm on S1). Ref. [58] postulated that the reduced stomatal size may be a mechanism of avoidance against the inhibitory effects of pollutants on physiological activities and a prompt response to external stimuli [59]. The reduced stomatal pore facilitates an elevation in the photosynthetic rate without excessive transpiration [60]. Ref. [53] asserted that this alteration in stomatal size is indicative of HM toxicity. On the other hand, this reduced stomatal size is accompanied by increased stomatal density, promoting plant survival in polluted environments. The elevated stomatal density is regarded as an indicator of the adaptive capacity to a polluted environment [48,61,62].

Regarding the *N. retusa* leaves, the transverse cross-sections frequently revealed swollen cells in the palisade parenchyma with a tendency toward complete cell collapse toward the lower surface. Nevertheless, a regular arrangement of cells was observed in the upper and lower epidermis. Occasionally, the epidermis was partially detached from the mesophyll. A conspicuous observation was made of the occurrence of numerous large idioblasts within the leaf mesophyll of the two species. Those appeared as dense dark spots; they were isolated, specialized, and stored cells. Their presence can be attributed to the plant’s adaptation to local microclimatic conditions or as a side effect of mining stress [63]. Ref. [61] suggests that these spots typically indicate the exposition of plants to stressful atmospheric conditions. The same author describes these spots as phenolic compounds. The increased accumulation of these compounds is considered one of the most common responses in stressed plants [64,65,66].

Under pollution, the two species displayed resilience to pollutants, enabling them to thrive in their natural environment. These findings support the research of [4,61], who found that *Trifolium montanum* and *Arabidopsis halleri* could reach maturity when facing changes in leaf structure caused by metal stress. In contrast, several studies have reported decreased growth in plant species, such as *Mangifera indica* [67] and Brassica juncea [68], due to metal pollution.

### 3.4. Correlation Between Anatomical Characteristics and Metal Contents

The correlation analysis highlights significant relationships between heavy metal concentrations (Zn, Cu, Pb, and Cd) and leaf morphology of *A. halimus* (Table 4). Zn positively correlates with leaf thickness (r = 0.860, *p* < 0.01) and upper epidermis thickness (r = 0.682, *p* < 0.05), while Pb correlates positively with stomatal density (r = 0.716, *p* < 0.05r) and negatively with mesophyll thickness (r = −0.825, *p* < 0.01), indicating structural adaptations to metal stress. Mesophyll thickness is strongly negatively correlated with Zn, Pb, and Lth (*p* < 0.01), suggesting trade-offs in leaf structure. Stomatal density increases with Zn and Pb, whereas stomatal size decreases, reflecting physiological adjustments to environmental stress. These findings demonstrate heavy metals’ impact on leaf structure and function.

The correlation results reveal significant relationships between heavy metal concentrations (Zn, Cu, Pb, and Cd) and morphological properties of *N. retusa* leaves, particularly the leaf thickness, mesophyll, upper and lower epidermal thickness, as well as stomatal characteristics (density and size) (Table 5). Cadmium (Cd) and lead (Pb) stand out with very strong, often negative correlations with parameters such as mesophyll thickness (r = −0.854, *p* < 0.01) and stomatal size (r = 0.877, *p* < 0.05), indicating a potential impact of these metals on the internal structure and physiological efficiency of the *Nitraria* leaves. Furthermore, positive correlations between Zn and the thickness of the lower epidermis suggest specific variations related to metal tolerance or accumulation. These findings highlight a complex and differentiated influence of heavy metals on the anatomical organization of leaves, potentially as a response to environmental stress.

The influence of HMs on the morpho-anatomical characteristics of many plant species has been investigated in numerous research studies, revealing significant relationships [69,70,71].

## 4. Conclusions

The study of two wild halophyte species, *N. retusa* and *A. halimus*, highlighted distinct physiological and anatomical responses to soil contamination by HMs. *A. halimus* accumulated higher levels of Zn and Cu in its leaves, while *N. retusa* preferentially stored Pb and Cd. Structural analysis revealed that metal exposure triggered the formation of idioblasts, specialized cells that may be involved in stress adaptation. Additionally, variations in leaf cell thickness and stomatal density were observed, indicating potential adjustments in water regulation and gas exchange to counteract metal-induced stress. The stomatal index provided further insights into these physiological modifications.

These results underscore the adaptive strategies that enable *N. retusa* and *A. halimus* to thrive in metal-polluted environments, demonstrating their resilience and ecological significance. Both species hold significant potential for application in environmental management. They could be used in phytoremediation programs to clean up metal-contaminated soils, thanks to their ability to tolerate and accumulate HMs. Additionally, their resilience to harsh conditions makes them suitable for soil stabilization in degraded or arid regions, helping combat desertification.

Future research could explore their role in the extraction of economically valuable metals (phytoextraction) and investigate the potential use of their biomass in industries such as bioenergy or compost production, provided that appropriate safety measures are taken. Further studies on the accumulation of other metals, additional biochemical and anatomical parameters, and their combined response to other stressors, like salinity or drought, would enhance our understanding of their ecological and practical applications.

## Figures and Tables

**Table 1:** Geographic localization and bioclimatic characteristics of different populations.

Populations	Code	Bioclimatic Zone	Variant	Rainfall (mm/year)	Altitude (m)	Longitude	Latitude
El-hamma	S1	Higher semi-arid	Mild winter	182	25	35°83′	10°64′
Tebelbou	S2	Higher semi-arid	Mild winter	234	29	33°84′	10°31′
Mareth	S3	Higher semi-arid	Cool winter	234	26	33°4′	10°24′
Matmata	S4	Inferior-arid	Mild winter	125	600	10°40′	33°28′

Bioclimatic zones are defined according to Emberger’s pluviothermic coefficient: Q2 = 2000 P/(M2 − m2), where P is the average of annual rainfall (mm), M is the mean of maximum temperature (K) for the warmest month, and m is the mean of minimum temperature (K) for the coldest month. P, M, and m calculated as the average of the period from 1953 to 2003.

**Table 2:** The occurrence of histological changes in leaves of *Atriplex halimus* and *Nitraria retusa.* Values are given as the mean *±* SD (*n* = 3). Values in each column followed by different letters are significantly different (*p* < 0.05).

Paramtres	Species	S4	S1
Leaf thickness (μm)	*Nitraria* *Atriplex*	397.5 ^a^ (±13.43) 355.83 ^b^ (±9.17)	281.25 ^b^ (±3.69) 471.66 ^a^ (±8.81)
Upper epidermal thickness (μm)	*Nitraria* *Atriplex*	12 ^a^ (±0.60) 14.25 ^b^ (±2.02)	9.66 ^b^ (±0.60) 20 ^a^ (±1.90)
Lower epidermal thickness (μm)	*Nitraria* *Atriplex*	12.70 ^b^ (±0.28) 9.06 ^a^ (±0.69)	16.7 ^a^ (±0.80) 8.79 ^a^ (±0.36)
Mesophyll (μm)	*Nitraria* *Atriplex*	374.16 ^a^ (±11.01) 448.33 ^a^ (±13.47)	252.5 ^b^ ± 3.19 265.41 ^b^ (±4.16)
Cribro-vascular Bundles diameter(μm)	*Nitraria* *Atriplex*	90 ^a^ (±7.20) 94.16 ^a^ (±11.01)	92.5 ^a^ (±12.87) 100 ^a^ (±13.05)
Stomatal density (/cm^2^)	*Nitraria* *Atriplex*	41.87 ^b^ (±0.85) 59.7 ^b^ (±3.57)	52.75 ^a^ (±1.70) 75.475 ^a^ (±1.92)
Stomatal size (μm)	*Nitraria* *Atriplex*	39 ^a^ (±2.16) 53.75 ^a^ (±1.7)	33.67 ^a^ (±1.04) 41.8 ^b^ (±3.22)

**Table 3:** Heavy metal concentration in leaves of *Atriplex halimus* and *Nitraria retusa* at various sites in Gabes. Values are given as the mean *±* SD (*n* = 3). Values in each column followed by different letters are significantly different (*p* < 0.05).

	Zn (mg kg^−1^)		Cu (mg kg^−1^)		Pb (mg kg^−1^)		Cd (mg kg^−1^)	
Sites	*Atriplex*	*Nitraria*	*Atriplex*	*Nitraria*	*Atriplex*	*Nitraria*	*Atriplex*	*Nitraria*
S1	183.33 a (±3.51)	112.16 a (±3.29)	54.93 ab (±5.1)	45.93 a (±4)	36.66 a (±10.5)	42.33 a (±3)	2.16 a (±0.08)	4.026 a (±0.06)
S2	174.5 a (±30.09)	110.16 a (±13.95)	56 a (±3.6)	50.33 a (±5.03)	21.33 b (±3.21)	35.33 ab (±4.72)	2.11 a (±0.1)	3.97 a (±0.26)
S3	164.66 a (±7.23)	97.66 a (±15)	56.26 a (±3.95)	50.9 a (±3.65)	16.66 b (±3.21)	25 bc (±5.56)	2.17 a (±0.18)	2.84 b (±0.09)
S4	96.36 b (±17.41)	84.83 a (±17)	47.66 b (±2.51)	26 b (±6.24)	10.66 b (±1.52)	20.93 c (±1.9)	1.9 a (±0.1)	2.43 c (±0.2)

**Table 4:** The values of Pearson’s correlation coefficient (*p* < 0.05) between heavy metals content in leaves of *A. halimus* and anatomical characters.

Variable	Zn	Cu	Pb	Cd	Lth	Uth	Low	Mes	Cvas	StoD	Stos
Zn	-										
Cu	0.663 *	-									
Pb	0.679 *	0.151	-								
Cd	0.423	0.251	0.361	-							
Lth	0.860 **	0.479	0.778 *	0.283	-						
Uth	0.682 *	0.215	0.688 *	0.182	0.859 **	-					
Low	−0.217	−0.362	−0.056	0.149	−0.392	−0.570	-				
Mes	−0.737 *	−0.358	−0.825 **	−0.297	−0.947 **	−0.850 **	0.448	-			
Cvas	0.221	0.011	0.250	0.334	0.126	0.202	0.179	−0.221	-		
StoD	0.702 *	0.472	0.716 *	0.590 *	0.733 *	0.728 *	−0.357	−0.795 *	0.417	-	
Stos	−0.725 *	−0.091	−0.744 *	−0.163	−0.849 **	−0.832 **	0.318	−0.842 **	−0.129	−0.547	-

Lth: leaf thickness; Uth: upper epidermal thickness. Low: low epidermal thickness. Mes: mesophyll thickness; Cvas: cribrovascular bundles diameter. StoD: stomatal density. Stos: stomatal size; *: significative at *p* ˂ 0.05; ** height significant at *p* < 0.01.

**Table 5:** The values of Pearson’s correlation coefficient (*p* < 0.05) between heavy metals content in leaves of *Nitraria retusa* and anatomical characters.

Variable	Zn	Cu	Pb	Cd	Lth	Uth	Low	Mes	Cvas	Stod	Stos
Zn	-										
Cu	0.399	-									
Pb	0.513	0.385	-								
Cd	0.683 *	0.605 *	0.766 **	-							
Lth	−0.322	−0.624 *	−0.79 **	−0.696 *	-						
Uth	−0.461	−0.625 *	−0.501	−0.767 **	0.654 *	-					
Low	0.659 *	0.651 *	0.677 *	0.742 **	−1.538	−0.656 *	-				
Mes	−0.653 *	−0.742 **	−0.854 **	−0.888 **	0.873 **	0.745 **	−0.783 **	-			
Cvas	0.071	0.091	0.214	0.185	−1.039	0.059	0.067	−0.115	-		
StoD	0.628 *	0.358	0.877 **	0.723 **	−0.838 **	−0.56	0.575	−0.833 **	−0.086	-	
Stos	−0.214	−0.162	−0.676 *	−0.542	0.792 **	0.488	−0.135	0.589 *	−0.028	−0.808 **	-

Lth: leaf thickness; Uth: upper epidermal thickness. Low: low epidermal thickness. Mes: mesophyll thickness; Cvas: cribrovascular bundles diameter. StoD: stomatal density. Stos: stomatal size; *: significative at *p* ˂ 0.05; ** height significant at *p* < 0.01.

## Data Availability

No new data were created or analyzed in this study. Data sharing is not applicable to this article.
